# Shear Stress Enhances the Paracrine-Mediated Immunoregulatory Function of Human Periodontal Ligament Stem Cells via the ERK Signalling Pathway

**DOI:** 10.3390/ijms23137119

**Published:** 2022-06-27

**Authors:** Ravipha Suwittayarak, Nuttha Klincumhom, Utapin Ngaokrajang, Worachat Namangkalakul, João N. Ferreira, Prasit Pavasant, Thanaphum Osathanon

**Affiliations:** 1 Center of Excellence for Regenerative Dentistry, Department of Anatomy, Faculty of Dentistry, Chulalongkorn University, Bangkok 10330, Thailand; 6378011232@student.chula.ac.th (R.S.); utapin.ng@gmail.com (U.N.); rumtdent@gmail.com (P.P.); 2 Dental Stem Cell Biology Research Unit, Department of Anatomy, Faculty of Dentistry, Chulalongkorn University, Bangkok 10330, Thailand; worachat.n@chula.ac.th (W.N.); thanaphum.o@chula.ac.th (T.O.); 3 Avatar Biotechnologies for Oral Health and Healthy Longevity Research Unit, Faculty of Dentistry, Chulalongkorn University, Bangkok 10330, Thailand; joao.f@chula.ac.th

**Keywords:** immunosuppression, IDO, TGF-β1, periodontal ligament stem cells, shear stress, T cells, mechanotransduction

## Abstract

Relevant immunomodulatory effects have been proposed following allogeneic cell-based therapy with human periodontal ligament stem cells (hPDLSCs). This study aimed to examine the influence of shear stress on the immunosuppressive capacity of hPDLSCs. Cells were subjected to shear stress at different magnitudes (0.5, 5 and 10 dyn/cm${}^{2}$). The expression of immunosuppressive markers was evaluated in shear stress-induced hPDLSCs using qRT-PCR, western blot, enzyme activity and enzyme-linked immunosorbent assays. The effects of a shear stress-derived condition medium (SS-CM) on T cell proliferation were examined using a resazurin assay. Treg differentiation was investigated using qRT-PCR and flow cytometry analysis. Our results revealed that shear stress increased mRNA expression of *IDO* and *COX2* but not *TGF-β1* and *IFN-γ*. IDO activity, kynurenine and active TGF-β1 increased in SS-CM when compared to the non-shear stress-derived conditioned medium (CTL-CM). The amount of kynurenine in SS-CM was reduced in the presence of cycloheximide and ERK inhibitor. Subsequently, T cell proliferation decreased in SS-CM compared to CTL-CM. Treg differentiation was promoted in SS-CM, indicated by *FOXP3*, *IL-10* expression and CD4${}^{+}$CD25${}^{hi}$CD127${}^{lo/-}$ subpopulation. In conclusion, shear stress promotes kynurenine production through ERK signalling in hPDLSC, leading to the inhibition of T cell proliferation and the promotion of Treg cell differentiation.

## 1. Introduction

Human periodontal ligament stem cells (hPDLSCs) can be isolated from the periodontal ligament, the connecting fibrous tissue between the tooth root and adjacent alveolar bone. To date, hPDLSCs have exhibited mesenchymal stem cell (MSC) characteristics owing to their potential for self-renewal and differentiation [1,2]. These cells have been proposed as a potential cell source for periodontal tissue regeneration due to their ability to differentiate and generate all periodontal tissue components after in vivo transplantation [3,4,5]. The therapeutic potential of hPDLSCs is indicated not only in the periodontal tissues but also in other tissues, such as the alveolar bone [6].

From an immunomodulatory standpoint, hPDLSCs possess striking features allowing them to escape immune recognition, inhibit activated immune cell function and regulate inflammatory cytokines during tissue regeneration [1,7,8,9,10,11,12,13]. These properties were initially recognised in MSCs that possess the ability to regulate the proliferation and differentiation of immune cells during an inflammatory response in order to promote tissue healing [14,15,16]. The significant immunomodulatory effect of paracrine immunosuppressive cytokines, including indoleamine 2,3-dioxygenase (IDO), hepatocyte growth factor (HGF), interleukin-10 (IL-10) and transforming growth factor beta1 (TGF-β1), has been reported to modulate immune cell behaviour.

Transforming growth factor beta 1 (TGF-β1) is constitutively expressed in MSCs and periodontal ligament (PDL) cells [10,17]. It is secreted as a latent form bounded to the cell membrane and extracellular matrix (ECM) proteins prior to activation [18,19]. In addition to regulation of tissue homeostasis, TGF-β1 can suppress T cell proliferation and activation [17,20,21] as well as promote the differentiation of regulatory CD4${}^{+}$ T (Treg) cell [22,23].

Indoleamin-2,3-dioxygenase (IDO) is an enzyme catabolising L-tryptophan into kynurenine. The depletion of tryptophan through the IDO–kynurenine pathway modulates immune response by inhibiting T cell proliferation and promoting Treg differentiation [24,25,26,27]. The role of IDO as an immune suppressor was shown to be activated in the presence of pro-inflammatory cytokines, for instance, IL-1β [28], IL-12 [29], Toll-like receptors (TLRs) [7,30], cyclooxygenase 2 (COX2)/prostaglandin E2 (PGE2) [31,32], interferon gamma (IFN-γ) [1,30,33] as well as TGF-β1 [34], leading to suppression of inflammation. Recently, our group showed that TLR3 induced the expression IFN-γ-independent IDO, resulting in inhibition of peripheral blood mononuclear cell (PBMC) proliferation and upregulation of a Treg-specific gene marker, *forkhead box P3* or *FOXP3* [7]. Additionally, mechanical stimuli have also been applied in vitro to regulate immunomodulatory properties of both PDL cells [35,36] and bone marrow-derived MSCs (BM-MSCs) [37]

PDL cells are considered as mechanosensory cells that can perceive and respond to mechanical stress driven by mastication, speech or orthodontic movement [38]. Several studies suggested that mechanical stimuli are important factors in regulating periodontal tissue homeostasis [39,40,41]. Previously, our group showed that intermittent compressive force regulated SOST/POSTN expression via the TGF-β1 signalling pathway in PDL cells [42].

In periodontal tissue, PDL cells are exposed to interstitial fluid shear stress during tooth movement [43,44]. Shear stress has been shown to play an essential role in cell behaviour in several cell types including embryonic stem cells [45], osteocytes [46], endothelial cells [47], MSCs [48] as well as PDL cells [43,49,50,51,52,53]. Previously, in vitro shear stress stimulation (3–15 dyn/cm${}^{2}$) was shown to modulate PDL cell properties such as osteogenic differentiation, cell proliferation and ECM remodelling [49,50,51,52]. Moreover, shear stress has the potential to enhance the immunosuppression of MSCs via COX2/PGE2 expression [37].

However, the influence of shear stress in triggering immunomodulatory properties of hPDLSCs has not been elucidated yet. Herein, we assess the effects of shear stress on the immunosuppressive properties of hPDLSCs. The influence of shear stress-activated immunosuppressive molecules in regulating CD4${}^{+}$ T cell proliferation and Treg differentiation was investigated. Our study suggests the use of mechanical stimuli of hPDLSCs as a promising approach to induce immunosuppression while targeting tissue regeneration with allogeneic therapies.

## 2. Materials and Methods

### 2.1. Isolation and Culture of hPDLSCs

Human PDL tissues were scraped from the middle third of the root surface obtained from normal healthy teeth that were scheduled to be extracted according to the treatment plan at the Department of Oral and Maxillofacial Surgery, Faculty of Dentistry, Chulalongkorn University. The human cell isolation protocol was approved by the Ethical Committee for Human Research (HREC-DCU 2022-010). The scraped PDL tissues from at least 3 patients were used for cell explant culture. The outgrown cells were maintained in Dulbecco’s modified Eagle medium (DMEM) (cat. No. 11885-084, Thermo Scientific, Waltham, MA, USA) containing 10% (*v*/*v*) fetal bovine serum (FBS) (cat. No. SV30160.03, Thermo Scientific), 1% (*v*/*v*) L-glutamine (Glutamax TM-1) (cat. No. 35050-061, Thermo Scientific), 1% (*v*/*v*) antibiotic–antimycotic (cat. No. 15240-062, Thermo Scientific) at 37 °C in a humidified 5% CO_2_ atmosphere. The culture medium was removed and changed every 2 days. Human periodontal stem cells (hPDLSCs) were subcultured using 0.25% trypsin/EDTA (cat. No. 25200-072, Thermo Scientific) until cells reached confluency. Cells in passages 3 through 7 were characterised and used in this study.

### 2.2. Flow Cytometry Analysis

For characterisation of hPDLSCs, cells were stained with PERCP-conjugated anti-human CD45 (cat. No. 21810455, ImmunoTools, Friesoythe, Germany), FITC-conjugated anti-human CD73 (cat. No. 212270733, ImmunoTools), FITC-conjugated anti-human CD90 (cat. No. ab11155, Abcam, Cambridge, UK) and PE-conjugated anti-human CD105 antibodies (cat. No. 21271054, ImmunoTools). For the Treg population, after indirect co-culture, all conditions of T cell culture were stained with VioBlue-conjugated anti-human CD45, VioGreen-conjugated anti-human CD4, VioBright 515-conjugated anti-human CD25 and PE-conjugated anti-human CD127 antibodies from the Treg Detection Kit (cat. No.130-122-994, MACS, San Diego, CA, USA). Mouse IgG1 PERCP, PE and FITC antibodies (MACS) were used as an isotype control. The stained cells were further detected by a flow cytometer (BD FACSCalibur and Cell Quest software, BD Bioscience, San Jose, CA, USA).

### 2.3. Induction of Osteogenic Differentiation

The cells were seeded into 24-well plates (cat. No. 142475, Thermo Scientific) at a density of $2\times 10^{4}$ cells/cm${}^{2}$ and cultured for 10–14 days in an osteogenic medium containing 10% FBS-DMEM supplemented with 50 μg/mL L-ascorbic acid (cat. No. A-4034, Sigma-Aldrich, St. Louis, MO, USA), 100 nM dexamethasone (cat. No. D8893, Sigma-Aldrich) and 5 mM β-glycerophosphate (cat. No. G9422, Sigma-Aldrich). The medium was changed every 2 days.

### 2.4. Induction of Adipogenic Differentiation

The cells were seeded into 24-well plates (Thermo Scientific) at a density of $2\times 10^{4}$ cells/cm${}^{2}$ and cultured for 16–20 days with an adipogenic medium containing 10% FBS-DMEM supplemented with 500 μmol 3-isobutyl-1-methylxanthine (IBMX) (cat. No. PH21124, Thermo Scientific), 1 μg/mL insulin from bovine pancreas, 100 μM indomethacin (cat. No. I7378, Sigma-Aldrich) that was kept at room temperature before use. The medium was changed every 2 days.

### 2.5. Alizarin Red S and Von Kossa Staining

For alizarin red S staining, the cells were fixed with cold methanol (cat. No. 100230, honey, Ulsan, Korea) for 10 min and washed with deionised (DI) water. Then, cells were stained with 1% alizarin red S (cat. No. A5533, Sigma-Aldrich) solution for 5 min at room temperature (RT). The samples were then rinsed with DI water gently and left to dry.

For von Kossa staining, cells were fixed with 4% (*v*/*v*) cold paraformaldehyde (AR1068, BOSTER, Pleasanton, CA, USA) in for 20 min, then rinsed in DI water. Silver nitrate (cat. No. 21572.188, VWR International GmbH, Vienna, Austria) at 5% (*w*/*v*) in DI water was added to the fixed cells for 30 min. The cells were exposed to a 100 w UV lamp for 10 min and left to dry. All samples were observed by inverted microscopy (Nikon ECLIPSE Ts2, Nikon, Melville, NY, USA).

### 2.6. Oil Red O Staining

The cells were fixed with 4% (*v*/*v*) formalin (cat. No. F-1268, Sigma-Aldrich) in DI water and gently washed with DI water. The samples were fixed with oil red O (cat. No. O0625, Sigma-Aldrich) solution in methanol (cat. No. 1.06009.2500, Merk, Germany) for 15 min. The samples were rinsed and DI water was replaced. All samples were then observed by inverted microscopy (Nikon ECLIPSE Ts2).

### 2.7. Isolation and Activation of CD4${}^{+}$ T Cells from PBMCs

Human PBMCs were isolated from the human buffy coat with permission from the Thai Red Cross Society. The protocols were approved by the Ethical Committee for Human Research (HREC-DCU-2022-010). The CD4${}^{+}$ T cell isolation was performed using Sepmate-50 (Stem Cell Technologies, Singapore). The isolated CD4${}^{+}$ T cells were maintained in RPMI 1640 (cat. No. 11835-030, Thermo Scientific) supplemented with 10% (*v*/*v*) FBS (Thermo Scientific), 1% (*v*/*v*) L-glutamine (Glutamax TM-1) (Thermo Scientific) and 1% (*v*/*v*) antibiotic–antimycotic (Thermo Scientific). For the T cell activation, the isolated CD4${}^{+}$ T cells were seed into 24-well plates (Thermo Scientific) at a density of 10${}^{6}$ cells/well and activated with 10% FBS-RPMI medium supplemented with 1 μg/mL CD3 (coated on 24-well plate overnight) (cat. No. 21850030, Immuno Tool), 1 mg/mL CD28 (cat. No. 302933, Biolegend) and 50 U/mL IL-2 (cat. No. 200-02, Peprotech) before the start of the conditioned medium treatment.

### 2.8. Shear Stress Stimulation

The hPDLSCs were seeded into 35 mm culture dishes (cat. No. 430165, Costar^®^ Corning) at a density of 4 × 10${}^{5}$ cells/well overnight. The culture medium was then changed to fresh medium. The hPDLSCs were subjected to different magnitudes of shear stress (0.5, 5 and 10 dyn/cm${}^{2}$) using a cone-shaped rotating disk for 3 h [54]. Thereafter, the hPDLSCs were cultured for 24 h in normal culture conditions before sample collection for mRNA, protein analysis and conditioned medium. For inhibitory experiments, the hPDLSCs were pretreated with 100 nM cycloheximide (CHX) (cat. No. C-0934, Sigma-Aldrich) or 1.5 nM ERK inhibitor (cat. No. 328006, Calbiochem, San Diego, CA, USA) for 1 h prior to shear stress stimulation. The hPDLSCs without shear stress stimulation and non-shear stress-induced hPDLSC-derived conditioned medium were used as the control and CTL-CM, respectively.

### 2.9. Cell Viability Assay

The Cell Counting Kit-8 (CCK-8) (cat. No. ab228554, Abcam, Cambridge^®^, UK) was used to detect the cell viability of hPDLSCs. After shear stress stimulation, the hPDLSCs were incubated with CCK-8 solution for 30 min. The 100 μL of secreted soluble formazan in the culture medium was put into 96-well plates and the absorbance was measured at 460 nm using a microplate reader (Synergy H1, Biotek multi-mode reader, Winooski, VT, USA) according to the manufacturer’s protocol. The data were calculated as the percentage of viable cells.

### 2.10. Immunofluorescent Staining

After shear stress stimulation, hPDLSCs were fixed with 4% (*v*/*v*) paraformaldehyde (BOSTER) in DI water and incubated with rhodamine–phalloidin (dilution 1:1000) (cat. No. ab235138, Abcam, Cambridge, UK). Stained cells were then counterstained with DAPI (dilution 1:2000) (TOCRIS bioscience, Bristol, UK). Then, 50% glycerol was added to the plate and stored at 4 °C. Apotome microscopy (Axio Observer Z1 and ZEN pro, ZEISS International, Oberkochen, Germany) was performed.

### 2.11. Conditioned Medium Preparation and Treatment

The conditioned medium was collected from hPDLSC cultures and kept at −80 °C before use. Prior to T cell culture experiments, frozen conditioned medium was thawed and lyophilised with a Tabletop Freeze Dryer (Medfuture Biotech, Jinan, China). Lyophilised conditioned medium was suspended with 10% FBS-RPMI medium and used for CD4${}^{+}$ T cell culture that was activated as previously described. All conditioned medium-treated T cells were collected for the T cell proliferation assay and investigation of Treg cell differentiation on day 3 and day 5 of the culture period, respectively.

### 2.12. T Cell Proliferation Analysis Using Resazurin Assay

For investigation of T cell proliferation, the activated CD4${}^{+}$ T cells were co-cultured with the conditioned medium for 3 days [7]. The CD4${}^{+}$ T cells were incubated with 7-hydroxy-10-oxidophenoxazin-10-ium-3-one, sodium (resazurin) (cat. No. R7017, Sigma-Aldrich) for 2 h. The fluorescence signal of secreted pink resorufin in culture medium converted from blue resazurin solution was measured using a microplate reader at excitation of 560 nm and emission of 590 nm and gain = 50. The data were presented as the percentage of proliferative cells.

### 2.13. Development of Regulatory T Cell (Treg) Cells

For induction of Treg cell differentiation, activated CD4${}^{+}$ T cells were exogenously treated with kynurenine (1–100 μM) (cat. No. K8625, Sigma-Aldrich), or cultured with hPDLSC-derived conditioned medium for 5 days before sample collection for qRT-PCR and flow cytometry analysis. The activated CD4${}^{+}$ T cells treated with 100 μM kynurenine were used as a positive control (or induced Treg).

### 2.14. RNA Isolation and Real-Time RT-PCR Analysis

Total RNA was extracted by using RiboExTM solution (cat. No. 301-902, GeneAll^®^, Seoul, Korea). The quality of RNA concentration was measured using a NanoDrop (Thermo Scientific). The amount of RNA was converted into complementary DNA (cDNA) using an ImProm-IITM Reverse Transcription System (cat. No. A3800, Promega, Madison, WI, USA). A FastStart Essential DNA Green Master kit was used for the real-time polymerase chain reaction process. The reaction was performed on a Bio-Rad PCR system (CFX Connect Real-Time System, Bio-Rad, Hercules, CA, USA). Cycling conditions were set at 95 °C for 30 s, followed by 45 cycles of 95 °C for 3 s and 60 °C for 30 s. Relative gene expression was calculated using the 2${}^{-\Delta \Delta \mathrm{CT}}$ method [55]. The expression value was normalised to the GAPDH expression value and the control. Oligonucleotide primers used in this study are shown in Table S1.

### 2.15. Enzyme-Linked Immunosorbent Assay (ELISA)

The supernatant and cell lysate were collected and extracted by RIPA buffer containing protease inhibitor buffer (cat. No. P8340, Sigma-Aldrich) after the shear stress experiment. Protein levels were measured using PGE2 (cat No. KGE004B, R&D system, Minneapolis, MN, USA), IFN-γ (cat. No. DY285-05, R&D system) and TGF-β1 (cat No. DY240-05, R&D system) following the manufacturer’s instructions.

### 2.16. IDO Activity Assay and Kynurenine Measurement

All the conditioned media were collected and prepared with IDO buffer containing 40 mM L-ascorbic acid (Sigma-Aldrich), 20 μM methylene blue (cat. No. MB-1, Sigma-Aldrich), 200 μg/mL catalase (cat. No. C9322, Sigma-Aldrich) and 800 μM of L-tryptophan solution (cat. T-8659, Sigma-Aldrich). In short, the samples were mixed with IDO buffer in a 1:1 ratio for 1 h at 37 °C in a humidified 5% CO_2_ atmosphere to convert tryptophan into kynurenine. To stop the reaction, 30% (*v*/*v*) trichloroacetic acid (TCA) in DI water (cat. No. T6399, Sigma-Aldrich) was added and incubated at 56 °C for 30 min. The sample was centrifuged at 13,000× *g* and added to 2% (*w*/*v*) Ehrlich reagent (cat. No. 39070, Sigma-Aldrich) in glacial acetic acid in a 1:1 ratio. For measurement of kynurenine product, 100 μL of all samples were mixed with 50 μL of 30% *v*/*v* TCA, then centrifuged at 8000× *g* for 5 min. Then, 75 μL of supernatant was equally mixed with 2.5% (*w*/*v*) Ehrlich reagent (Thermo Scientific) in glacial acetic acid. All samples were then read at 492 nm using a microplate reader [1]. The recombinant kynurenine (Sigma-Aldrich) was used as a standard in this experiment.

### 2.17. Western Blot Assay

The protein from shear stress-induced hPDLSCs was extracted by RIPA buffer containing protease inhibitor buffer (cat. No. P8340, Sigma-Aldrich). The concentration of lysate proteins was measured with a BCA protein assay (PierceTM BCA detection reagent, cat No. 23228 and 23224, Thermo Scientific). Lysate proteins were separated on 12% SDS-polyacrylamide gel and transferred to a nitrocellulose membrane. The membrane was placed in a solution containing a monoclonal antibody to rabbit-anti actin (cat. No. A2066, Sigma-Aldrich), rabbit anti-TGF-β1 (cat No. ab92486, Abcam), rabbit anti-ERK1/2 (T202/Y204, Cell Signaling) or rabbit anti-phosphorylated ERK1/2 (cat. No. 137F5, Cell Signaling Technology) antibodies. Then, the membranes were developed with the horseradish peroxidase-linked antibody (cat No. 7074S, Cell signaling Technology). The membranes were visualised and exposed to chemiluminescence (SuperSignal^®^ West Pico Chemiluminescent Substrate, cat No. 34577, Thermo Scientific) and an image analyser (GE Healthcare, Pittsburgh, PA, USA), respectively. The band density was measured using ImageJ software. The band density was normalised to band density of actin and to the control.

### 2.18. Statistical Analysis

All data are presented as mean ± standard deviation (SD). One way-ANOVA and the Mann–Whitney U test were using for comparisons between groups. A *p*-value below 0.05 was considered significant. The analysis was performed by using the statistical software GraphPad Prism version 8 (GraphPad software, San Diego, CA, USA). At least three replicates from different donors were performed for each experiment.

## 3. Results

### 3.1. Shear Stress Enhanced the Expression of Immunosuppressive Regulators

Isolated hPDLSCs were characterised and positive for MSC-specific markers and the ability to differentiate into osteoblastic and adipogenic lineages (Figure S1). The effect of shear stress on viability of hPDLSCs was determined using the CCK-8 assay. We found that all magnitudes of shear stress (0.5–10 dyn/cm${}^{2}$) had no significant effect on cell viability, which was indicated by the mitochondrial activity of hPDLSCs (Figure S2).

**Figure 1:**
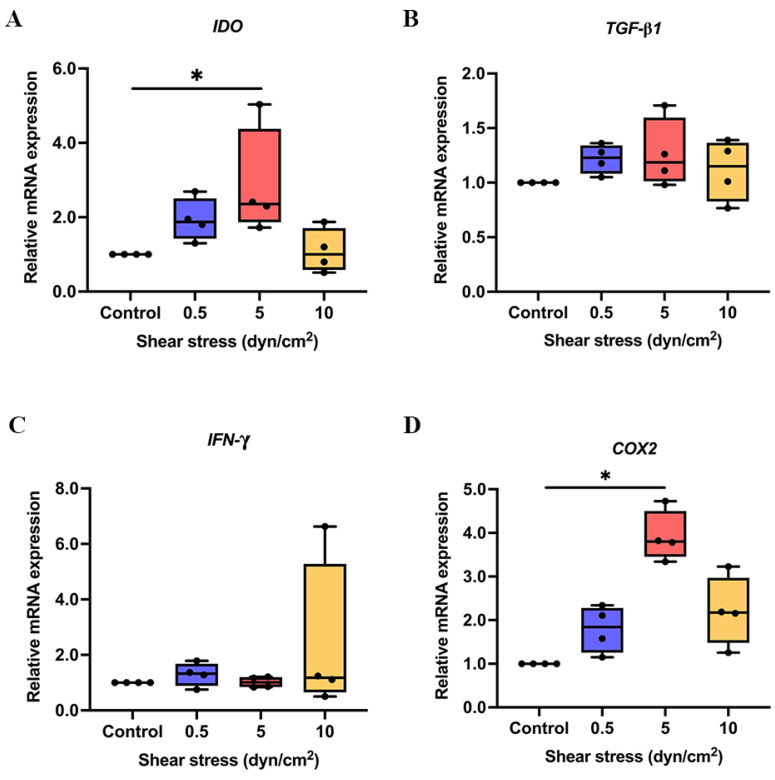
The mRNA expression of immunosuppressive regulators in shear stress-induced hPDLSCs. After shear stress stimulation, the relative mRNA expression of *IDO* (**A**), *TGF-β1* (**B**), *IFN-γ* (**C**) and *COX2* (**D**) was detected using qRT-PCR. * *p* < 0.05 vs. control group.

To determine whether shear stress stimulates the expression of immunomodulatory regulators in hPDLSCs, cells were subjected to shear stress at 0.5, 5, 10 dyn/cm${}^{2}$ for 3 h and continuously cultured afterwards up to 24 h. Cells were harvested for mRNA expression analysis of immunomodulatory regulators, including *IDO*, *TGF-β1*, *COX2* and *IFN-γ*. The stress at 5 dyn/cm${}^{2}$ significantly increased the gene expression of IDO (Figure 1A) and *COX2* (Figure 1D) in hPDLSCs, while there is no significant difference in the expression of *IFN-γ* (Figure 1C) and *TGF-β1* (Figure 1B).

**Figure 2:**
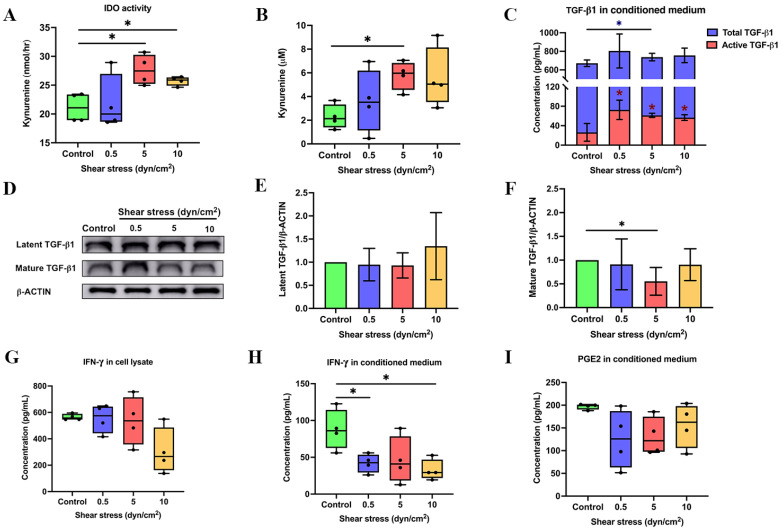
Protein expression of immunosuppressive regulators in shear stress-induced hPDLSCs. Shear stress promoted IDO activity (**A**) and the kynurenine product (**B**) in hPDLSCs. Shear stress increased the secretion of active TGF-β1 in PDLSC-derived conditioned medium (**C**). TGF-β1 protein expression in hPDLSCs was determined by Western blot analysis (**D**). The quantitative analysis of Western blot band intensity of latent TGF-β1(**E**) and active TGF-β1 (**F**). The concentration of IFN-γ in cell lysate (**G**) and conditioned medium (**H**) and the amount of COX2-independent PGE2 were determined by ELISA assay (**I**). * *p* < 0.05 vs. control group.

We further investigated the activity of IDO and kynurenine products, which were measured in all conditioned media after shear stress stimulation (Figure 2A,B). The IDO activity increased in shear stress-derived conditioned medium (SS-CM) at 5 and 10 dyn/cm${}^{2}$ compared to the non-shear stress-derived conditioned medium, also referred to as a control (CTL-CM) (Figure 2A). Subsequently, the amount of kynurenine product in SS-CM at 5 dyn/cm${}^{2}$ was significantly higher than CTL-CM (Figure 2B). With regard to *TGF-β1*, the protein expression of active TGF-β1 was determined in the conditioned medium. Although the secretion of total TGFβ1 was increased in 5 dyn/cm${}^{2}$ shear stress, active TGF-β1 in SS-CM was increased at all shear stress magnitudes (0.5, 5 and 10 dyn/cm${}^{2}$) compared to the CTL-CM (Figure 2C). In contrast, compared to control, shear stress decreased the active form of TGF-β1 in cell lysates at 5 dyn/cm${}^{2}$ yet had no effect on the latent form of TGF-β1 (Figure 2D–F). The amount of IFN-γ in cell lysate and conditioned medium was measured using an ELISA assay. Interestingly, this assay showed that the amount of cell-bound IFN-γ in all magnitudes of shear stress was not different compared to control (Figure 2G). In contrast, the amount of secreted IFN-γ decreased at 0.5 and 10 dyn/cm${}^{2}$ (Figure 2H). As for the *COX2* expression, we analysed COX2-dependent PGE2 synthesis in hPDLSCs with different magnitudes of shear stress. Outcomes showed no difference in the PGE2 product of all groups of SS-CM compared to CTL-CM (Figure 2I). Our results are supported by previous literature [49,50,51,52] and indicated that a 5 dyn/cm${}^{2}$ magnitude of shear stress can optimally enhance IDO, kynurenine and TGF-β1 gene/protein expression.

### 3.2. Shear Stress Enhanced the Product of IDO Activity via ERK1/2 Signalling Pathway

**Figure 3:**
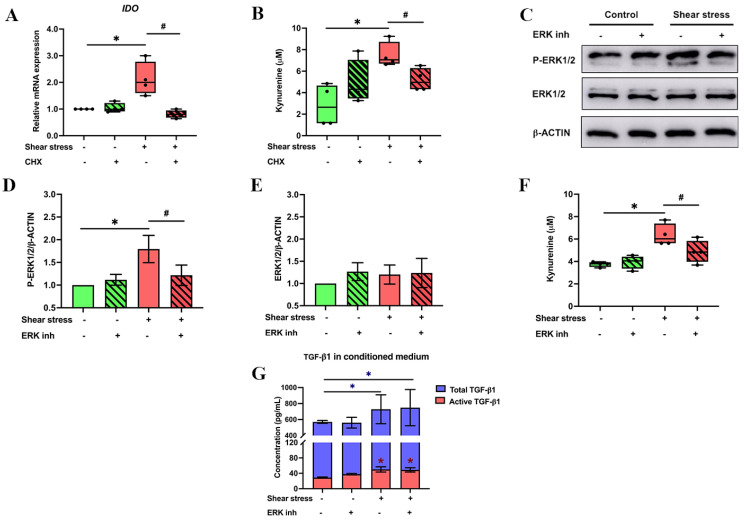
Shear stress enhanced IDO expression and IDO-catabolised kynurenine product in hPDLSCs via ERK1/2 signalling pathway. After shear stress stimulation (at 5 dyn/cm${}^{2}$), hPDLSCs treated with CHX were examined for *IDO* mRNA expression and kynurenine product (**A**,**B**). The activity of ERK1/2 was examined by Western blot analysis (**C**). The quantitative analysis of Western blot band intensity showed that ERK inhibitor attenuated P-ERK1/2 (**D**), but not ERK1/2 expression in shear stress-induced hPDLSCs (**E**). The addition of an ERK inhibitor inhibits the effect of shear stress-induced kynurenine secretion (**F**). However, shear stress did not affect TGF-β1 secretion (**G**). * *p* < 0.05 vs. non-shear stress without ERK inhibitor group. # *p* < 0.05 vs. control.

To investigate the regulatory mechanism by which shear stress enhances IDO expression and the amount of kynurenine in hPDLSC-derived conditioned medium, cells were pretreated with protein synthesis inhibitor cycloheximide (CHX) for 1 h before shear stress stimulation (5 dyn/cm${}^{2}$). As a result, CHX attenuated the expression of *IDO* and the amount of kynurenine when compared to control (Figure 3A,B). This result suggested the involvement of intermediate molecules in the regulatory mechanism of shear stress-induced *IDO* gene expression in hPDLSCs.

Next, the role of extracellular signal-regulated kinase 1/2 (ERK1/2) was demonstrated to be associated with the activation of immunosuppressive properties of human dendritic cells [51]. To investigate whether shear stress regulates the IDO–kynurenine and TGF-β1 secretion via ERK1/2 activation, hPDLSCs were pretreated with ERK inhibitor for 1 h and subsequently were placed under shear stress at 5 dyn/cm${}^{2}$ for 3 h. In the control condition, cells received shear stress for 3 h without pretreatment with an ERK inhibitor. The effect of shear stress on protein expressions of ERK1/2 and phosphorylated ERK1/2 (P-ERK1/2) was determined by Western blot. Shear stress induced the phosphorylation of ERK1/2 of hPDLSCs, which was abolished by the ERK inhibitor (Figure 3C–E). The effect of shear stress on the amount of kynurenine in hPDLSCs was also attenuated in the presence of ERK inhibitor (Figure 3F), but not TGF-β1 and its active form (Figure 3G). Therefore, shear stress enhanced the kynurenine secretion in hPDLSCs via the activation of the ERK1/2 signalling pathway.

### 3.3. Shear Stress-Derived Conditioned Medium (SS-CM) Suppressed T Cell Proliferation

**Figure 4:**
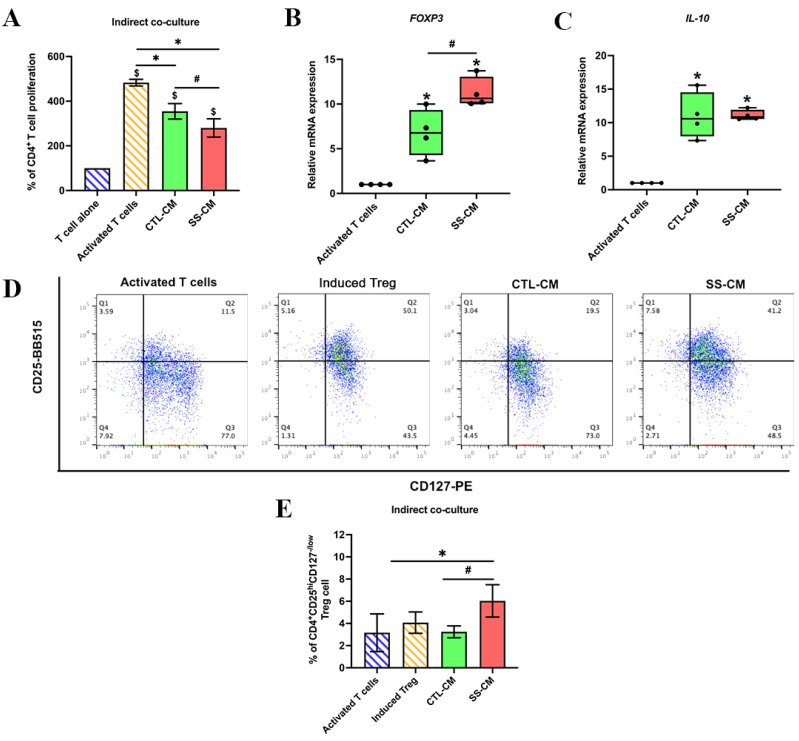
Shear stress attenuated CD4${}^{+}$ T cell proliferation and promoted Treg differentiation. All hPDLSC-derived conditioned media were indirectly co-cultured with CD4${}^{+}$ T cells. The proliferation of CD4${}^{+}$ T cells was assessed using a resazurin assay. SS-CM decreased the percentage of T cell proliferation (**A**). The mRNA expressions of *FOXP3* (**B**) and *IL-10* (**C**) were upregulated in SS-CM-treated CD4${}^{+}$ T cells. The flow cytometry analysis showed an increase in the percentage of CD4${}^{+}$CD25${}^{hi}$CD127${}^{lo/-}$ Treg population in SS-CM compared to CTL-CM and activated T cells (**D**,**E**). $ *p* < 0.05 vs. T cell alone group, * *p* < 0.05 vs. activated T cells group, # *p* < 0.05 vs. CTL-CM group.

To evaluate the inhibitory effect of SS-CM on the proliferation of CD4${}^{+}$ T cells from PBMCs, the conditioned medium was lyophilised and suspended with a fresh 10% FBS-RPMI medium. Isolated T cells were activated with CD3, CD28 and IL-2, which are co-stimulatory signals from antigen-presenting cells (APCs) for activation and expansion of T cells [52]. The activated T cells were treated with SS-CM for 3 days as the optimal timing [7]. The CTL-CM was used as a control. Suspended cells in each condition were observed and collected for a T cell proliferation assay. After T cell activation, the activated T cells remarkably formed clusters, increased cell size and increased in number (Figure S3A–D). T cell proliferation was significantly decreased after treatment with CTL-CM and SS-CM compared to activated T cells without conditioned medium treatment. In addition, the proliferation of T cells was significantly lower in SS-CM than in CTL-CM (Figure 4A). Our results suggested that a conditioned medium derived from hPDLSCs inhibited the proliferation of T cells, and this effect can be enhanced by shear stress stimulation.

### 3.4. Shear Stress-Derived Conditioned Medium (SS-CM) Induced Regulatory T Cell Differentiation

To further investigate whether SS-CM induces the development of regulatory T (Treg) cells, activated T cells were cultured with SS-CM for 5 days. The Treg cells were characterised by Treg-specific gene markers, *FOXP3* and *IL-10*. The results showed that the mRNA expression of *FOXP3* significantly increased in SS-CM compared to activated T cells and CTL-CM (Figure 4B). The expression of *IL-10* in CTL-CM and SS-CM was upregulated compared to activated T cells. However, the mRNA expression of *IL-10* was not different between CTL-CM and SS-CM (Figure 4C). A previous study indicated that CD4${}^{+}$CD25${}^{hi}$CD127${}^{lo/-}$ are potent purity markers of the functional Treg population [53,56,57]. Our study showed that SS-CM increased the percentage of CD4${}^{+}$CD25${}^{hi}$CD127${}^{lo/-}$ Treg cells compared to activated T cells and CTL-CM (Figure 4D,E). Therefore, these outcomes suggested that a conditioned medium derived from shear stress-induced hPDLSCs enhances mRNA expression of Treg-specific markers (*FOXP3* and *IL-10*) and increases the Treg cell population.

## 4. Discussion

Here, our study showed that shear stress enhanced the secretion of TGF-β1 and IDO-catabolised kynurenine in a conditioned medium derived from shear stress-induced hPDLSCs. These conditioned media potentially inhibited CD4${}^{+}$ T cell proliferation while promoting CD4${}^{+}$CD25${}^{hi}$CD127${}^{lo/-}$ Treg differentiation.

TGF-β1 is an immunosuppressive molecule that induces Treg differentiation and inhibits T cell proliferation [17,20,21,22]. Our data showed that the shear stress at 5 dyn/cm${}^{2}$ enhanced the secretion of TGF-β1 and the active form of TGF-β1. These findings confirmed that shear stress might be one factor in regulating TGF-β1 activation, similar to platelets [58]. Kynurenine is a product from IDO-metabolised tryptophan. IDO and kynurenine are important factors regulating the T cell function [25]. The decreasing of tryptophan by IDO activity inhibited T cell growth [24]. Moreover, increased kynurenine induced immune cell apoptosis and Treg differentiation [26,27,59]. Interestingly, our study also showed that shear stress activated the expression and activity of the IDO enzyme. Although the amount of kynurenine detected in SS-CM was much lower than that of the exogenous kynurenine experiment (Figure S4B), SS-CM markedly suppressed T cell immune activity. A previous study showed that the aryl hydrocarbon receptor (AHR) on T cells is needed for kynurenine-induced Treg cell differentiation. Moreover, the AHR can be increased when treated with TGF-β1, promoting Treg cell differentiation [60]. Our data suggest that an increased amount of TGF-β1 in SS-CM may amplify the effect of kynurenine on immune suppression. However, further studies on how shear stress-activated IDO and TGF-β1 regulate immunosuppressive properties of hPDLSCs may unveil other mechanisms.

Additionally, shear stress-induced IDO mRNA expression and kynurenine product were inhibited by CHX, the protein synthesis inhibitor, suggesting the involvement of intermediate activators during shear stress stimulation. Studies indicate that IFN-γ and PGE2 are potent inducers to activate IDO expression [31,32,61]. We speculated that shear stress might regulate immunosuppressive properties of hPDLSCs via IFN-γ or PGE2-independent IDO activation. This speculation is based on our previous study showing that TLR3 enhanced the immunosuppressive capacity of hPDLSCs by suppressing proliferation of T cells and promoting expression of *FOXP3* mRNA via IFN-γ-independent IDO activation [7]. Moreover, TGF-β1 had been shown to induce IDO expression in dendritic cells via the P(I)3K signalling pathway, resulting in a positive feedback loop for generating TGF-β1 and IDO [34,62]. We speculate that shear stress may also regulate IDO expression of hPDLSCs via the TGF-β1 signalling pathway.

We further investigated the mechanotransduction-mediated signalling pathway of hPDLSCs after shear stress stimulation. ERK1/2, as a subset of the MAPK signalling pathway, has been reported to be involved in shear stress-mediated mechanotransduction, leading to regulation of the expression of target genes [52,63]. Our study found that shear stress activated the ERK1/2 signalling pathway by increasing the phosphorylation of ERK1/2. The activity of ERK1/2 usually results in increased immunosuppressive effects of dendritic cells via increased TGF-β1 secretion [56]. Our study showed no difference in TGF-β1 secretion after adding ERK inhibitor, indicating that shear stress-induced TGF-β1 secretion in hPDLSCs was not regulated by ERK1/2 activity. Additionally, P38, as a subset of MAPK kinase, is upstream of IDO expression when activated by viral stimulation [64]. Herein, our study showed that kynurenine product in SS-CM was attenuated in the presence of an ERK inhibitor. These data suggested that shear stress enhances kynurenine production in hPDLSCs via the ERK1/2 signalling pathway.

Immunosuppressive properties of PDL cells can be activated by pro-inflammatory cytokines. Conditioned medium from PDL cells exposed to IFN-γ and TLR3 could inhibit T cell proliferation and induce *FOXP3* mRNA expression [1,7]. Here, our study demonstrated that shear stress potentially enhanced the immunosuppressive properties of hPDLSCs by suppressing T cell proliferation and stimulating Treg cell differentiation. Treg cell features were investigated by gene expression of specific markers (*FOXP3* and *IL10*) and protein expression of specific cell surface markers (CD4${}^{+}$CD25${}^{hi}$CD127${}^{lo/-}$) [65,66,67]. Moreover, flow cytometry of viable CD4${}^{+}$CD25${}^{hi}$CD127${}^{lo/-}$ Treg cells and sublocalisation of FOXP3 protein via western blot can be further analysed to investigate Treg cell activity [68,69].

**Figure 5:**
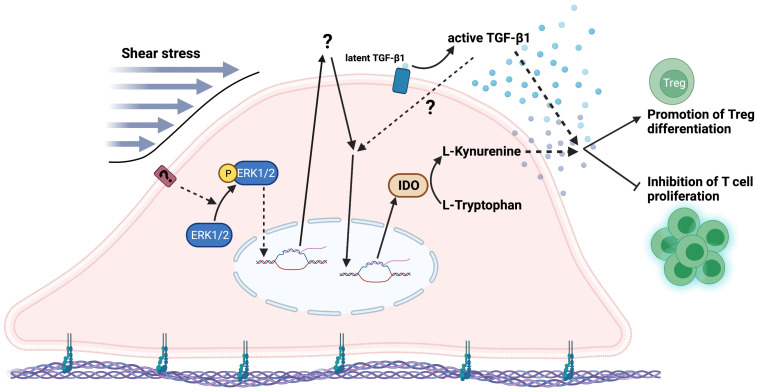
Schematic diagram of shear stress activates an immunosuppressive ability of hPDLSCs via ERK-induced IDO and total and active TGF-β1. Shear stress enhances IDO-dependent kynurenine in hPDLSCs via ERK1/2 activation and increases total and active TGF-β1 in the extracellular matrix or conditioned medium. Increased kynurenine and TGF-β1 secretion inhibit the CD4${}^{+}$ T cell proliferation and promote Treg cell differentiation. Created with Biorender.com.

In conclusion, our findings demonstrated that shear stress enhanced kynurenine in hPDLSCs via the ERK1/2 signalling pathway. In response to shear stress, hPDLSCs secreted active TGF-β1 and kynurenine, thereby suppressing T cell proliferation and promoting Treg differentiation (Figure 5). Our findings contribute to a better understanding of the immunosuppressive properties of hPDLSCs in response to mechanical stimuli such as the ones generated during tooth movement. We believe that the paracrine-mediated immunoregulatory function of hPDLSCs may be a promising cell-free approach for clinical applications, especially for the case of allogeneic cell therapy.

## Data Availability

The data presented in this study are available on request from the corresponding author.

## Supplementary Materials

The following supporting information can be downloaded at: https://www.mdpi.com/article/10.3390/ijms23137119/s1.

## Abbreviations

AHR	Aryl hydrocarbon receptor
BCA	Bicinchoninic acid
CHX	Cycloheximide
COX2	Cyclooxygenase 2
CTL-CM	Non-shear stress-induced hPDLSC-derived conditioned medium
DI	Deionised water
ELISA	Enzyme-linked immunosorbent assay
ERK1/2	Extracellular signalling-regulated kinase1/2
FOXP3	Forkhead box P3
GAPDH	Glyceraldehyde-3-phosphate dehydrogenase
hPDLSCs	Human periodontal ligament stem cells
IDO	Indoleamine 2,3dioxygenase
IFN-γ	Interferon gamma
IL-1β	Interleukin-1 beta
IL-2	Interleukin-2
IL-12	Interleukin-12
IL-10	Interleukin-10
Kyn	Kynurenine
MSCs	Mesenchymal stem cells
PBMCs	Peripheral blood mononucleated cells
P-ERK1/2	Phosphorylated extracellular signalling-regulated kinase ½
PGE2	Prostaglandin E2
PI	Protease inhibitor
P(I)3K	Phosphoinositide 3-kinase
RIPA	Radioimmunoprecipitation assay
RT	Room temperature
SS-CM	Shear stress-induced hPDLSC-derived conditioned medium
TCA	Trichloroacetic acid
TLRs	Toll-like receptors
Treg	Regulatory T cell
TGF-β1	Transforming growth factor beta 1
