# Supplementary material for: Shear Stress Enhances the Paracrine-Mediated Immunoregulatory Function of Human Periodontal Ligament Stem Cells via the ERK Signalling Pathway
Source: Int J Mol Sci. 2022 Jun 27;23(13):7119. doi: 10.3390/ijms23137119 (PMC9266779; doi:10.3390/ijms23137119)
Supplement: Supplementary file 1 [file ijms-23-07119-s001.zip › ijms-1768612-supplementary.pdf]

**Table S1.** Primer sequences

| Gene name | Sequence 5’-3’ |
|---|---|
| *GAPDH* | (F) CACTGCCAACGTGTCAGTGGTG |
| | (R) GTAGCCCAGGATGCCCTTGAG |
| *COX-2* | (F)GCCATGGGGTGGACTTAAATCAT |
| | (R)CAGGGACTTGAGGAGGGTAGATC |
| *IDO* | (F) CATCTGCAAATCGTGACTAAG |
| | (R) GTTGGGTTACATTAACCTTCCTT |
| *IL-10* | (F) TGCTCTTGCAAAACCAAACCA |
| | (R) TCGAAGCATGTTAGGCAGGTT |
| *IFN-γ* | (F) CTAGGCAGCCAACCTAAGCA |
| | (R) CAGGGTCACCTGACACATTC |
| *FOXP3* | (F) GATGGTACAGTCTCTGGAGCAGC |
| | (R) GTAGGGTTGGAACACCTGCTGG |
| *TGF-β1* | (F) AAAGATGGAGAGAGGACTGCG |
| | (R) AGTGCCCAAGGTGCTCAAT |

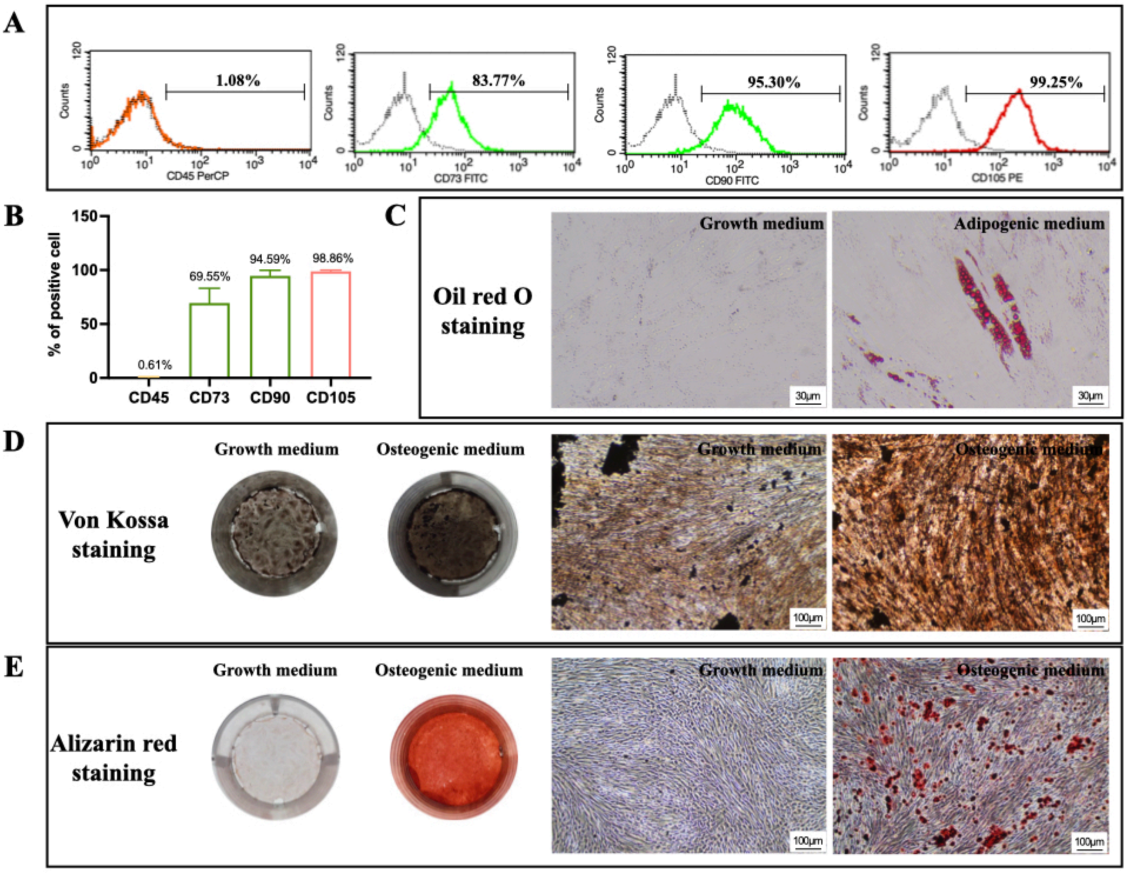


**Figure S1.** The characterization of human periodontal ligament stem cells (hPDLSC). The expression of CD73, CD90, and CD105 as positive MSCs markers and CD45 as negative markers was confirmed by using flow cytometry (**A**-**B**). The potential of differentiation into multilineage was determined for the adipogenic and osteogenic lineages. The hPDLSC-derived adipocyte was induced in adipogenic medium, compared to growth medium (**C**). The hPDLSC-derived osteoblast was induced in osteogenic medium, compared to growth medium (**D**-**E**). Scale bar = 30 μM, 100 μM.

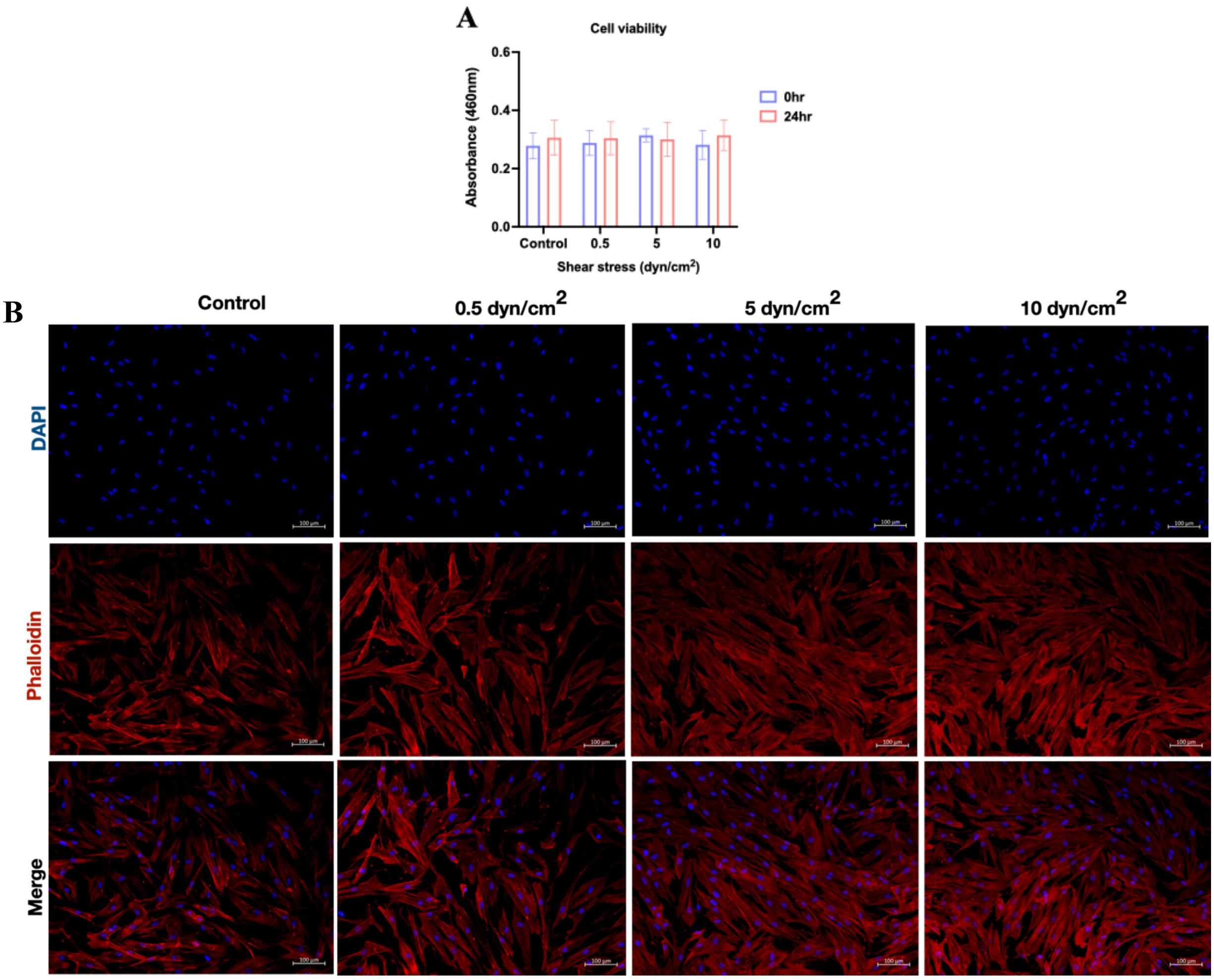


**Figure S2.** The effect of shear stress on morphology and viability of hPDLSC. Cell viability was measured by CCK-8 assay. The different shear stress-induced hPDLSC showed no difference in cell viability compared to control at 24 h (**A**). Cell morphology of shear stress-induced hPDLSC at different magnitudes (0.5, 5, and 10 dyn/cm$^2$) was observed using immunofluorescent staining of phalloidin-positive actin cytoskeleton (**B**). Scale bar = 30 μM, 100 μM.

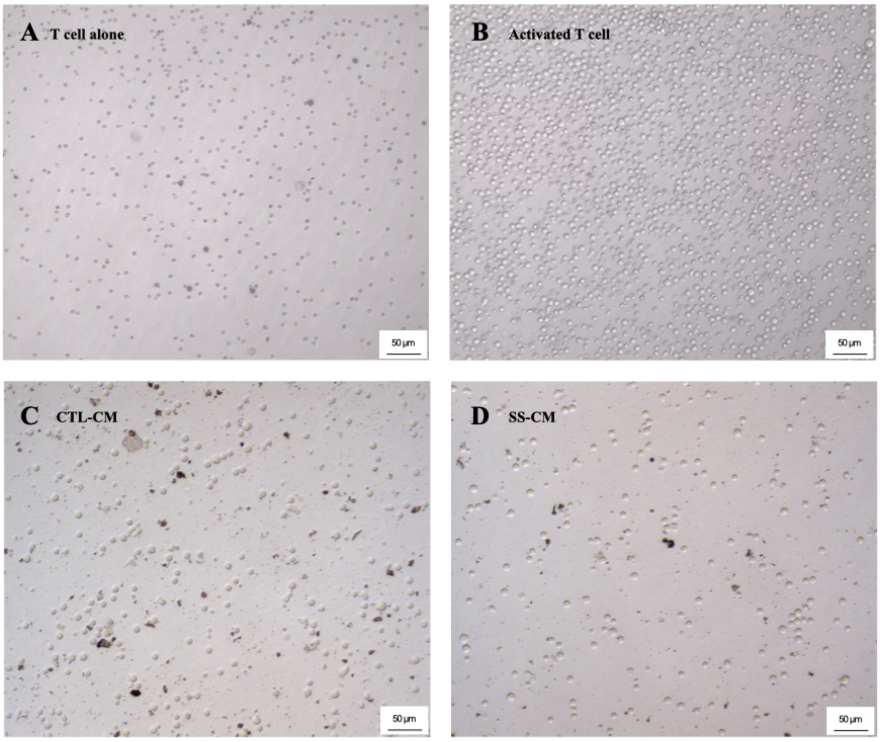


**Figure S3.** The morphology of PBMC-derived T cells. Inverted light microscopy imaging showed a small number of culture naïve or non-activated T cells in culture (**A**). An increased number of activated CD4$^+$ T cells after activation (**B**). The number of activated T cells was attenuated in T cells treated with CTL-CM (**C**) and SS-CM (**D**). Scale bar = 100 µM.

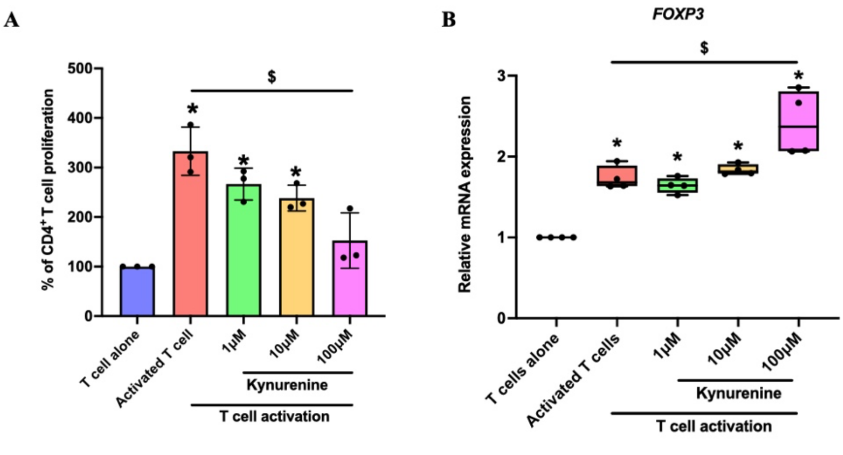


**Figure S4**. The effect of exogenous kynurenine on T cell proliferation and FOXP3 mRNA expression in activated T cells. Cell proliferation analysis using resazurin assay showed that 100 µm of exogenous kynurenine significantly decreased the percentage of T cell proliferation (**A**). While it significantly increased the FOXP3 mRNA expression compared to that of activated T cells (**B**). *p<0.05 vs T cell alone group. $p<0.05 vs activated T cells group
